# African Swine Fever Virus, Tanzania, 2010–2012

**DOI:** 10.3201/eid1812.121083

**Published:** 2012-12

**Authors:** Gerald Misinzo, Christopher J. Kasanga, Chanasa Mpelumbe–Ngeleja, Joseph Masambu, Annette Kitambi, Jan Van Doorsselaere

**Affiliations:** Sokoine University of Agriculture, Morogoro, Tanzania (G. Misinzo, C.J. Kasanga);; Central Veterinary Laboratory, Dar es Salaam, Tanzania (C. Mpelumbe-Ngeleja, J. Masambu);; Kilombero District Council, Ifakara, Tanzania (A. Kitambi);; and Higher Institute for Nursing and Biotechnology, Roeselare, Belgium (J. Van Doorsselaere)

**Keywords:** Asfarviridae, African swine fever virus, viruses, phylogeny, genotyping, persistent circulation, outbreak, Tanzania

**To the Editor:** African swine fever (ASF) is a highly contagious and deadly hemorrhagic disease of domestic pigs caused by African swine fever virus (ASFV), a double-strand DNA virus of the family *Asfarviridae* and genus *Asfivirus* (*1*). Twenty-two ASFV genotypes (I–XXII) have been identified on the basis of nucleotide sequencing of the variable 3′-end of the *B646L* gene encoding the major capsid protein p72 (*2*,*3*).

Historically, all ASFV p72 genotypes have been circulating in eastern and southern Africa, and genotype I has been circulating in Europe, South America, the Caribbean, and western Africa (*2*,*3*). Spread of ASFV beyond traditional geographic boundaries occurred with incursion of p72 genotype II into the Republic of Georgia and its subsequent spread into Armenia, Azerbaijan, and Russia (*4*,*5*) and incursion of genotype IX into western Africa (*6*). ASFV circulating in Tanzania has p72 genotypes X, XV, and XVI (*7*–*10*). We describe incursion and persistent circulation in Tanzania of a highly virulent p72 genotype II ASFV that is identical to the Georgia 2007/1 isolate in the 3′-end of the *B646L* gene.

An outbreak of ASF in domestic pigs occurred in November 2010 in the Kyela District of the Mbeya region in southwestern Tanzania, which coincided with another outbreak in a neighboring district of Karonga in northern Malawi (Figure, panel A). ASF continued to spread from Mbeya and ultimately reached the neighboring region of Iringa (Ludewa District) in February 2011 through feeding of pigs with swill from Mbeya. By March 2011, ASF had spread to Chunya, Ileje, Mbarali, Rungwe, and Tukuyu districts within Mbeya. The disease spread within the region because of the lack of zoosanitary measures and illegal movement of animals despite the quarantine in place. An outbreak on 1 farm in the Temeke District of the Dar es Salaam region in eastern Tanzania occurred in March 2011 after a farmer obtained pig stock from Mbeya. No further spread of the disease in Dar es Salaam was observed after early diagnosis, removal of affected pigs, and zoosanitary measures.

**Figure:**
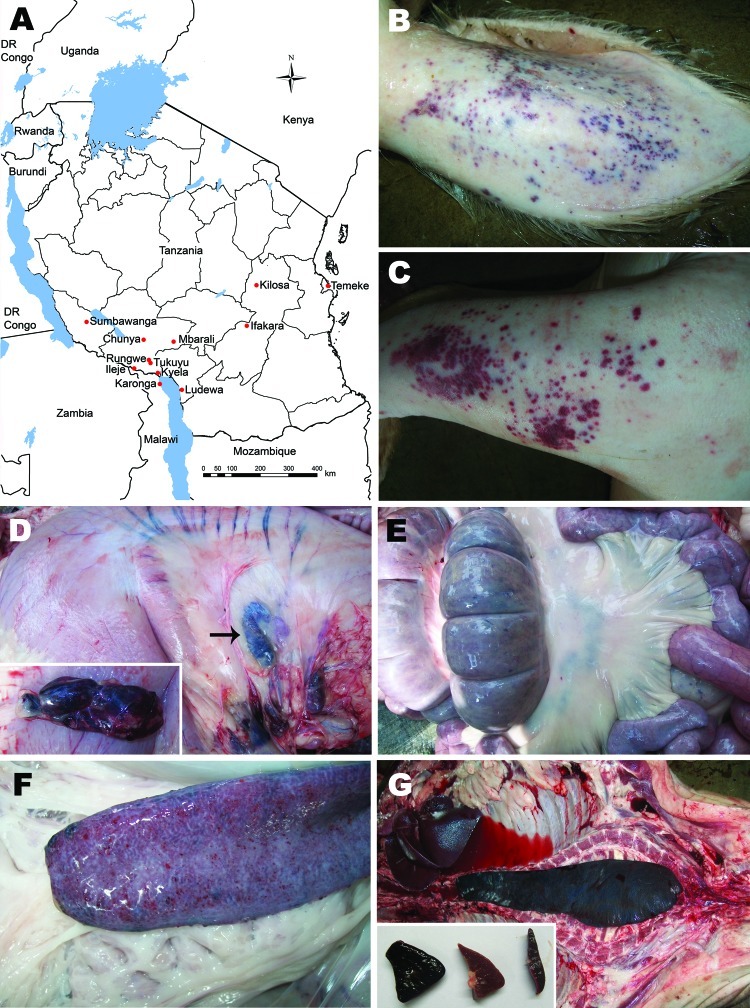
A) Locations in which African swine fever outbreaks occurred during 2010–2012. B–G) Postmortem lesions observed in slaughtered pigs at the Ifakara slaughterhouse of Kilombero District. Postmortem lesions include cutaneous hemorrhage on the medial side of the pinna (B) and forelimb above the carpal joint (C); hemorrhagic gastrohepatic lymph node (arrow and insert) (D), intestines (E) and spleen (F); and splenomegaly (G). Insert in panel G indicates portions of spleens obtained from different animals showing rounding of edges of the spleen caused by splenomegaly (spleen on the left and center) compared with normal edges of the spleen (spleen on the right). DR Congo, Democratic Republic of the Congo.

In October 2011, the disease spread to the Sumbawanga District of the Rukwa region through feeding of swill and illegal movement of animals. ASF was reported in February 2012 in Ifakara in the Kilombero District in the Morogoro region, and in July 2012 in the Kilosa District within this region. The disease spread into Kilombero District after 1 farmer purchased pigs for stock from the Iringa region. As of July 2012, ASF was reported again in the Mbeya and Iringa regions, from which it had been eliminated. This unique ASF outbreak in Tanzania persistently circulated for more than a year; previous outbreaks have been sporadic and resolved after shorter durations (*8*–*10*).

Mortality rates of 100% caused by ASF were recorded in domestic pigs of all ages in all outbreak areas. Affected pigs showed pyrexia and anorexia, dragged their hind legs, and then showed recumbence. In addition, affected animals had severe cutaneous hemorrhages, especially on medial and lateral sides of the pinna, forelimbs above the carpal joint, facial region, scrotum, and mammary glands (Figure, panels B and C). Postmortem lesions included darkening and enlargement of the spleen, severe hemorrhages of mesenteric and gastrohepatic lymph nodes, and hemorrhagic enteritis (Figure, panels D–G).

DNA was extracted from spleens of animals that either died of the disease or were killed at slaughterhouses during 2010–2012. The variable 3′-end of the *B646L* (p72) gene was amplified by using p72U/p72D primers (*2*) and subjected to automated dideoxynucleotide cycle sequencing by using Big Dye Terminator Cycle Sequencing Kit Version 3.1 (Applied Biosystems, Foster City, CA, USA) in a 24-capillary DNA sequencer (Genetic Analyzer 3500 xL; Applied Biosystems).

All obtained ASFV p72 nucleotide sequences (GenBank accession nos. JX391987 [TAN/10/Kyela], JX391988 [TAN/10/Tukuyu], JX391989 [TAN/11/Chunya], JX391990 [TAN/11/Ludewa], JX391991 [TAN/11/Temeke], JX391992 [TAN/12/Ifakara]) were 100% identical. BLAST (http://blast.ncbi.nlm.nih.gov/Blast.cgi) analysis of 2010–2012 ASFV p72 nucleotide sequences from Tanzania in GenBank showed 100% nucleotide identity with the Georgia 2007/1 ASFV isolate (GenBank accession no. FR682468) at nt positions 103,594–104,070.

The Georgia 2007/1 ASFV isolate was detected in Georgia in 2007 and has caused ASF outbreaks in Armenia, Azerbaijan, and Russia (*5*). This ASFV isolate belongs to p72 genotype II (*5*) and clusters with ASFV isolates from Mozambique, Zambia, Madagascar, Mauritius, and Georgia (*2*,*3*,*7*). Although the 2010–2012 outbreak in Tanzania coincided with the outbreak in Malawi in 2010, no ASFV belonging to p72 genotype II has been described in Malawi. ASFV isolates from the 2010 outbreak in Malawi should be sequenced to establish their relatedness to ASFV isolates from the 2010–2012 outbreak in Tanzania and determine an epidemiologic link between these outbreaks. Incursion and persistent circulation of a highly virulent p72 genotype II ASFV identical to the Georgia 2007/1 isolate has implications for transboundary spread of ASF.
